# Deciphering Genetic Architecture of Adventitious Root and Related Shoot Traits in *Populus* Using QTL Mapping and RNA-Seq Data

**DOI:** 10.3390/ijms20246114

**Published:** 2019-12-04

**Authors:** Pei Sun, Huixia Jia, Yahong Zhang, Jianbo Li, Mengzhu Lu, Jianjun Hu

**Affiliations:** 1 State Key Laboratory of Tree Genetics and Breeding, Key Laboratory of Tree Breeding and Cultivation of State Forestry and Grassland Administration, Research Institute of Forestry, Chinese Academy of Forestry, Beijing 100091, China; spfate@126.com (P.S.); huixia__jia@126.com (H.J.); lumz@caf.ac.cn (M.L.); 2 Experimental Center of Forestry in North China, Chinese Academy of Forestry, Beijing 102300, China; 3 Collaborative Innovation Center of Sustainable Forestry in Southern China, Nanjing Forestry University, Nanjing 210037, China

**Keywords:** *Populus*, adventitious root trait, shoot trait, QTL, RNA-Seq

## Abstract

Understanding the genetic architecture of adventitious root and related shoot traits will facilitate the cultivation of superior genotypes. In this study, we measured 12 adventitious root and related shoot traits of 434 F_1_ genotypes originating from *Populus deltoides* ‘Danhong’ × *Populus simonii* ‘Tongliao1’ and conducted an integrative analysis of quantitative trait locus (QTL) mapping and RNA-Seq data to dissect their genetic architecture and regulatory genes. Extensive segregation, high repeatability, and significant correlation relationship were detected for the investigated traits. A total of 150 QTLs were associated with adventitious root traits, explaining 3.1–6.1% of phenotypic variation (PVE); while 83 QTLs were associated with shoot traits, explaining 3.1–19.8% of PVE. Twenty-five QTL clusters and 40 QTL hotspots were identified for the investigated traits. Ten QTL clusters were overlapped in both adventitious root traits and related shoot traits. Transcriptome analysis identified 10,172 differentially expressed genes (DEGs) among two parents, three fine rooting and three poor-rooting genotypes, 143 of which were physically located within the QTL intervals. K-means cluster and weighted gene co-expression network analysis showed that *PtAAAP19* (*Potri.004G111400*) encoding amino acid transport protein was tightly associated with adventitious roots and highly expressed in fine-rooting genotypes. Compare with ‘Danhong’, 153 bp deletion in the coding sequence of *PtAAAP19* in ‘Tongliao1’ gave rise to lack one transmembrane domain, which might cause the variation of adventitious roots. Taken together, this study deciphered the genetic basis of adventitious root and related shoot traits and provided potential function genes for genetic improvement of poplar breeding.

## 1. Introduction

Forest trees constitute the largest pool of biomass carbon in the terrestrial ecosystem, providing great ecological and economic values [1]. Plant biomass production and distribution depend on the interactions between below-ground root and above-ground shoot. Root absorbs water and nutrients to offer growth substances for carbon fixation process in shoot leaves, while shoots provide mechanical support and transfer pathway for organic substances [2]. The biomass partitioning patterns between root and shoot are related to plant growth rate, life habitats, nutrient density, and environmental adaptability [3,4,5]. Vegetative propagation is an important reproductive pattern in many woody species, and adventitious root formation is a limiting step in vegetative propagation [6]. Enhancing the ability to regenerate the adventitious root can promote the wide cultivation of elite genotypes. Therefore, dissecting the genetic basis of adventitious root and shoot traits will accelerate plant breeding progress for cultivating the genotypes with high biomass and reproduction capability.

Poplar is a fast-growing tree species that represents one of the most appealing sources of renewable biomass feedstock [7]. Adventitious root and shoot development and biomass partitioning from morphological characteristics have been investigated in different poplar species under contrast environment conditions [3,8,9,10]. Moreover, some genes, such as *ethylene response factor* (*PtaERF003*), *WUSCHEL-related homeobox 5a* (*PtoWOX5*) and *type-B cytokinin response regulator* (*PtRR13*), involved in the auxin-dependent pathway have been reported to play important roles during adventitious root and shoot development in poplar [11,12,13]. Despite these advances, the molecular regulation mechanism of root and related shoot traits has not been comprehensively dissected. Root architecture and shoot traits are complex traits and controlled by multiple genes with a small genetic effect [14,15]. Quantitative trait locus (QTL) mapping is a powerful tool for the identification of complex traits-associated genes, which might possess low mean effect, epistatic interaction, or be from redundant families, being difficult to bring out using classical loss-of-function approaches [16]. Many studies have utilized QTL mapping to dissect growth-related traits in different species, such as maize, wheat, wild barley, potato, and *Arabidopsis thaliana* [17,18,19,20,21].

In poplar, several studies have analyzed below-ground and above-ground biomass allocation at the genome level under different growth conditions. The biomass distribution patterns have been confirmed to be under genetic control in F_2_ and BC_1_ population between *Populus trichocarpa × Populus deltoides* [22]. Rae et al. [1] investigated QTLs controlling adventitious root and shoot growth traits under ambient and elevated CO_2_, and identify three areas of the genome determining root growth in response to elevated CO_2_. The QTLs associated with carbon allocation and partitioning have been identified in *Populus* under two nitrogen conditions, and the majority of these QTLs are specific to one of the two conditions, indicating significant nitrogen-dependent genetic control [23]. In a study of the genetic architecture of shoot-root covariation of *Populus euphratica*, 15 and four heterochronic QTLs (hQTLs) were detected to mediate the forms of shoot and taproot growth, respectively; 11 pleiotropic hQTLs determining the covariation of shoot and taproot growth were identified by a bivariate mapping model [24]. However, our current knowledge of genetic underpinnings that govern adventitious root architecture and related shoot traits is far from complete.

To increase understanding of coordination of adventitious root and related shoot traits, we undertook a genetic basis dissection of poplar adventitious root architecture and related shoot traits in hydroponic culture, using a high-density linkage mapping with a full-sib family from *Populus deltoides* ‘Danhong’ × *Populus simonii* ‘Tongliao1’ which have contrast biological characteristics in regard to adventitious rooting ability and shoot growth traits [25]. This study aimed to identify putative genes controlling these traits in poplar through the integration of QTL mapping and transcriptome analysis. This study will provide fundamental insights into the genetic architecture of important traits for poplar.

## 2. Results

### 2.1. Analysis of Adventitious Root and Related Shoot Traits in the F_1_ Population

**Figure 1:**
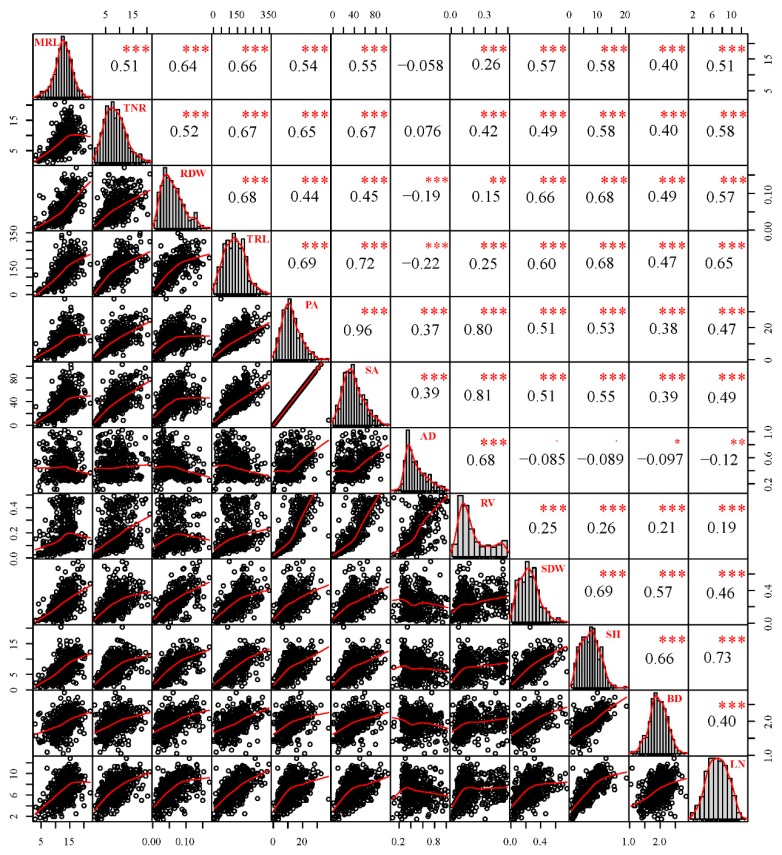
Scatter plots and correlations among adventitious root and related shoot traits of the F_1_ population. * *p* < 0.05; ** *p* < 0.01; *** *p* < 0.001; AD—average diameter; SA—surface area; SH—shoot height; BD—basal diameter; SDW—shoot dry weight; RV—root volume; LN—leaf number of shoot; PA—projected area; TRL—total number of root; TNR—total number of root; MRL—maximum root length.; RDW—root dry weight.

**Figure 2:**
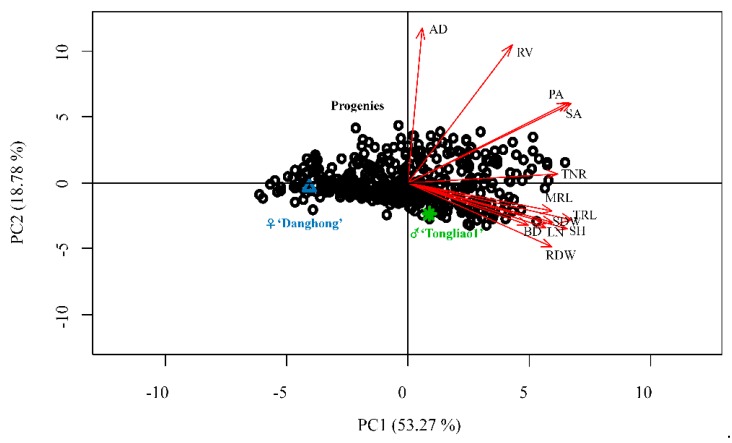
Principal component analysis of adventitious root and related shoot traits of the F1 population. PC1—principal component 1; PC2—principal component 2.

A total of 12 adventitious root and related shoot traits including maximum root length (MRL), total root length (TRL), total number of root (TNR), root dry weight (RDW), projected area (PA), surface area (SA), average diameter (AD), root volume (RV), shoot dry weight (SDW), shoot height (SH), basal diameter (BD), and leaf number of shoot (LN) were measured in two parents (*P. deltoides* ‘Danhong’ and *P. simonii* ‘Tongliao1’) and their 434 F_1_ hybrid genotypes. The hybrid parents exhibited a difference in adventitious root and related shoot traits, which contributed to distinct separation in the F_1_ population. Adventitious root primordia and stirring bud of over 90% of the genotypes were formed after 15 days of hydroponic culture. For the two parents, the development time of adventitious root primordia formation and bud stirring in ‘Danhong’ was later than in ‘Tongliao1’. Significant differences between the 12 analyzed traits except for AD, SDW, and BD between the two parents revealed their large genetic difference (Table 1). The maximum and minimum values in the population exceeded those in the parents, indicating transgressive inheritance. All the traits exhibited normal distribution based on absolute kurtosis and skewness value being less than 1 (Table 1). Correlation patterns among the 12 adventitious root and related shoot traits are displayed in Figure 1. Different degrees of significant positive correlation were detected among MRL, TNR, RDW, TRL, PA, SA, RV, SDW, SH, BD, and LN. A negative correlation was detected between AD and the other seven traits (MRL, RDW, TRL, SDW, SH, BD, and LN). The largest positive correlation occurred between PA and SA, followed by SA and RV. RV obtained the highest variation coefficient among the traits. All the shoot traits were positively correlated with the root traits except AD, and SH had the highest correlation with the root traits. The MRL, TNR, and RV had no significant differences among blocks, whereas the remaining traits exhibited significant differences among blocks (Table S1). The clonal repeatability for adventitious root traits ranged from 0.76 for AD to 0.89 for TNR, PA, and SA. Estimates of clonal repeatability for related shoot traits ranged from 0.72 for BD to 0.87 for LN (Table 1). These correlation and repeatability results indicated that genotypes with more below-ground adventitious roots tended to have larger above-ground shoot biomass. Principal component analysis (PCA) was applied to further explore the relationships among the traits within the F_1_ population. As expected, all the traits displayed a similar correlation extent and type pattern. Two groups of variables were identified as principal component 1 (PC1) and principal component 2 (PC2), which explained 53.27% and 18.78% of the total variation, respectively (Figure 2). All traits were totally positively weighed for PC1. TNR, PA, SA, AD, and RV were positively weighed for PC2, whereas MRL, RDW, TRL, SDW, SH, BD, and LN were negatively related to PC2. Notably, the two parents were separated from each other in PCA analysis.

### 2.2. QTL Mapping and Candidate Gene Identification

**Figure 3:**
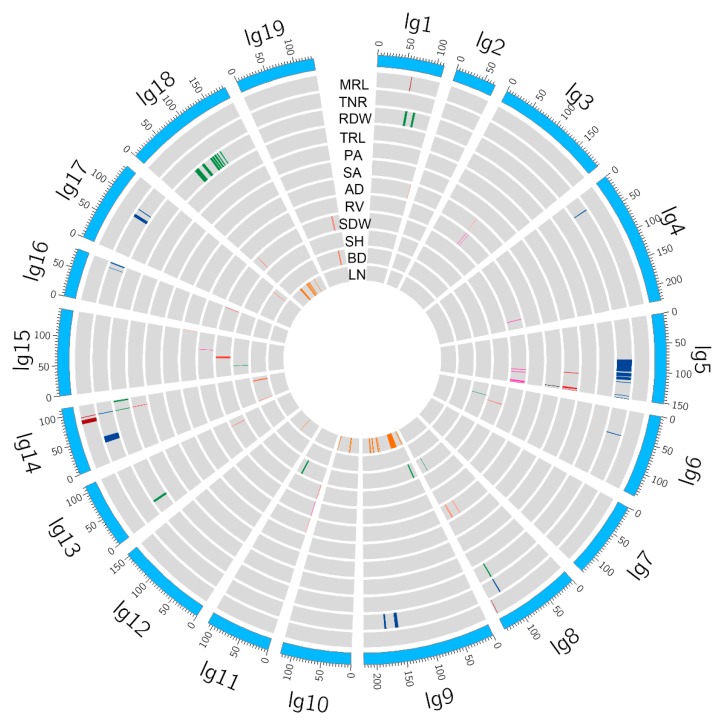
Circos plot exhibited quantitative trait locus (QTLs) of adventitious root and related shoot traits in the genetic map. The outside circle represented the linkage group of genetic map. The QTL loci of (MRL), total root length (TRL), total number of root (TNR), root dry weight (RDW), projected area (PA), surface area (SA), average diameter (AD), root volume (RV), shoot dry weight (SDW), shoot height (SH), basal diameter (BD), and leaf number of shoot (LN) arranged from the first circle to the twelfth circle.

A sum of 150 QTLs distributed on 14 linkage groups (LGs) were detected for eight adventitious root traits, and 365 candidate genes were identified within the corresponding QTL intervals (Figure 3, Table S2). Among them, LG5 had the largest number of 43 QTLs, followed by LG18 and LG14. The phenotypic variance explanation (PVE) ranged from 3.1% to 6.1%, and the LOD score varied from 3.0 to 5.95. Twelve QTLs were identified for MRL with three on LG1, two on LG8, and seven on LG14. The PVE for MRL ranged from 4.4% to 6.1%, and the LOD score ranged from 4.20 to 5.95. The distinctive numbers of QTLs for other adventitious root traits were as follow: TNR (43 QTLs), RDW (49 QTLs), TRL (1 QTL), PA (11 QTLs), SA (2 QTLs), AD (11 QTLs), and RV (21 QTLs). A total of 83 QTLs affecting four shoot traits distributed on 15 LGs and 275 candidate genes were identified in QTL intervals (Figure 3, Table S2). LG9 obtained the largest number of QTLs, followed by LG18 and LG10. The PVE of these QTLs ranged from 3.1% to 19.8%, with an LOD score of 3.00–5.36. The numbers of QTLs associated with each shoot traits were as follow: SDW (10 QTLs), SH (7 QTLs), BD (12 QTLs), and LN (44 QTLs).

### 2.3. QTL Clusters and Hotspots

Classical quantitative genetics hypothesize that the presence of QTL clusters and hotspots is ascribed to the very close linkage of genes in certain regions of the genome [26,27]. The QTL cluster refers to multiple different traits related to a QTL, and the QTL hotspot refers to multiple QTLs of the same trait distributed within the 20 cM region [28]. In this study, 25 QTL clusters were identified on eight LGs: 14 QTL clusters for adventitious root traits; one QTL cluster for shoot traits; 10 QTL clusters for both adventitious root traits and related shoot traits (Table 2). The QTL clusters on LG5 were associated with most traits (RV, TNR, PA, and SA), while LG18 contained a maximum of nine QTL clusters related to RDW and LN.

A sum of forty QTL hotspots was identified for investigated traits except for TRL (Table 3). LG5 had eight QTL hotspots associated with TNR, PA, SA, and RV. Twenty-seven QTL hotspots were identified for adventitious root traits on ten LGs, and 13 QTL hotspots were discovered for shoot traits on eight LGs. Among QTL hotspots for adventitious root traits, TNR obtained a maximum of ten QTL hotspots on LG1 (1), LG6 (1), LG8 (1), LG16 (1), LG17 (1), LG9 (2), and LG5 (3). For shoot traits, LN possessed a maximum of eight QTL hotspots on LG9 (5), LG10 (1), and LG18 (2). Overlapping confidence intervals between different traits corresponding QTLs were also detected in these QTL hotspots.

### 2.4. RNA-Seq Profiling

To explore the molecular basis of morphology difference of adventitious root, RNA-Seq analysis was conducted in two parents (’Danhong’ and ‘Tongliao1’), three genotypes with poor rooting ability (named B group) and three genotypes with fine rooting ability (named G group). A total of 184.45 Gb raw reads were generated, with an average length of 150 bp (Table S3). After removing low-quality sequences and trimming adapter, the output clean reads of all samples ranged from 5.89 Gb to 9.76 Gb representing about 14- to 23-fold of the *P. trichocarpa* genome size. The average GC content and Q30 value of clean reads were 44.10% and 94.97%, respectively. Briefly, 120.7 million of all reads (84.4%) were mapped to the *P. trichocarpa* genome. About 98.4 million of the reads (81.5%) could be uniquely mapped to the genome, and 3.5 million of the reads (2.9%) were multiple mapped (Table S4). Pearson’s correlation tests showed that ‘Danhong’ was the most distinct compared to ‘Tongliao1’ with correlation coefficient ranging from 0.699 to 0.866 (Figure S1A). Three genotypes (G1, G2, and G3) in the G group showed quite similar with correlation coefficient ranging from 0.953 to 0.995; Three genotypes (B1, B2, and B3) in the B group were also closely related with correlation coefficient ranging from 0.905 to 0.993. Principal component analysis revealed that the three biological replicates of each genotype were clearly assigned together (Figure S1B). Taken together, these results indicated fine biological replicates and distinct differences among two parents, B group and G group.

### 2.5. Differentially Expressed Gene (DEG) Analysis

**Figure 4:**
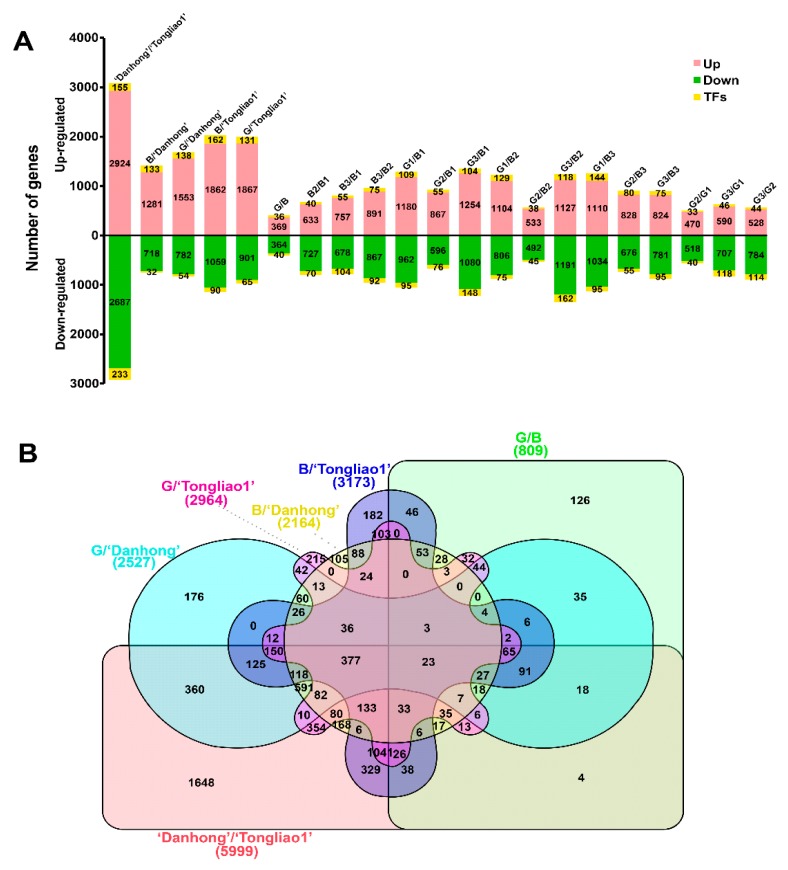
Statistics of differentially expressed genes (DEGs) derived from pairwise comparisons among eight genotypes (‘Danhong’, ‘Tongliao1’, three poor-rooting genotypes (B1, B2, and B3), and three fine-rooting genotypes (G1, G2, and G3)). (**A**) The number of upregulated and downregulated genes in 21 pairwise comparison sets. (**B**) Venn diagram of DEGs showing commonly or uniquely regulated genes at different comparison sets.

**Figure 5:**
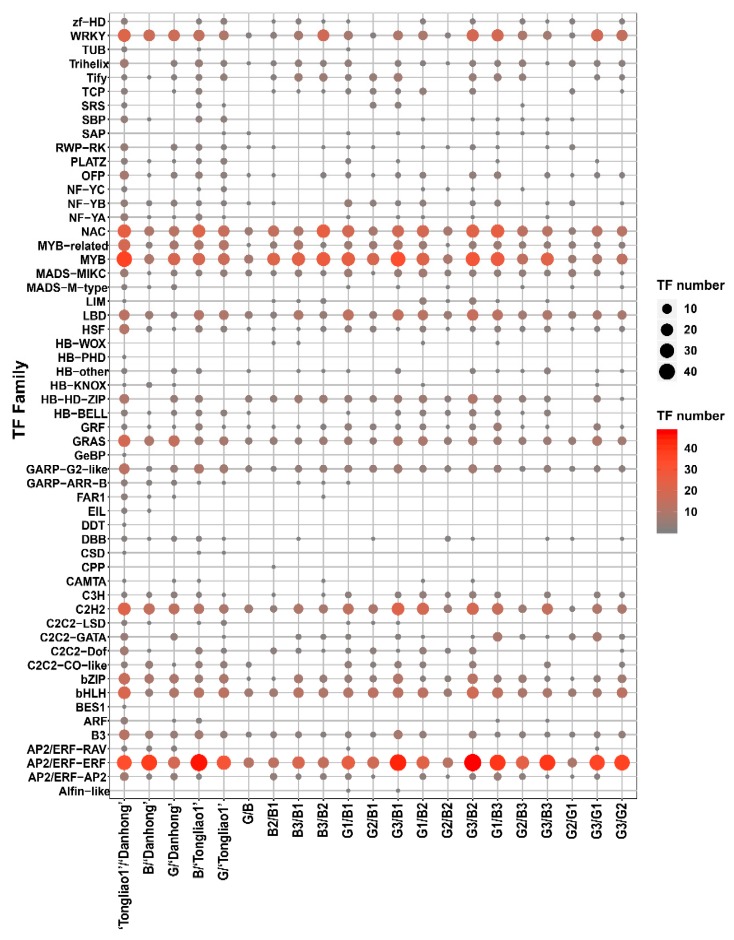
Summary of differentially-expressed transcription factors (TFs) in the 21 pairwise comparison sets.

A total of 10,172 DEGs were identified in 21 pairwise comparison sets (Figure 4A, Table S5,6). There were 5999 DEGs, including 3079 upregulated genes and 2920 downregulated genes between ‘Danhong’ and ‘Tongliao1’, 1648 DEGs of which were unique in this comparison set (Figure 4). Moreover, 809 DEGs containing 405 upregulated genes and 404 downregulated genes were detected between the G group and B group, 126 DEGs of which were unique in this comparison set. Among these DEGs, 807 transcription factors (TFs) were identified and assigned to 56 TF families (Figure 5). The *MYB*, *AP2/ERF,* and *bHLH* exhibited higher expression in ‘Danhong’ than ‘Tongliao1’, whereas the *C2H2*, *GRAS,* and *NAC* showed the opposite tendency. The *WRKY*, *NAC*, *MYB*, *C2H2*, and *AP2/ERF-ERF* exhibited a significant difference in 21 pairwise comparison sets (Figure 5). Furthermore, 143 DEGs were located within the QTL intervals, including *MYB*, *RLK* protein kinase, and others (Table S7).

### 2.6. Clustering Analysis of DEGs in Different Genotypes

**Figure 6:**
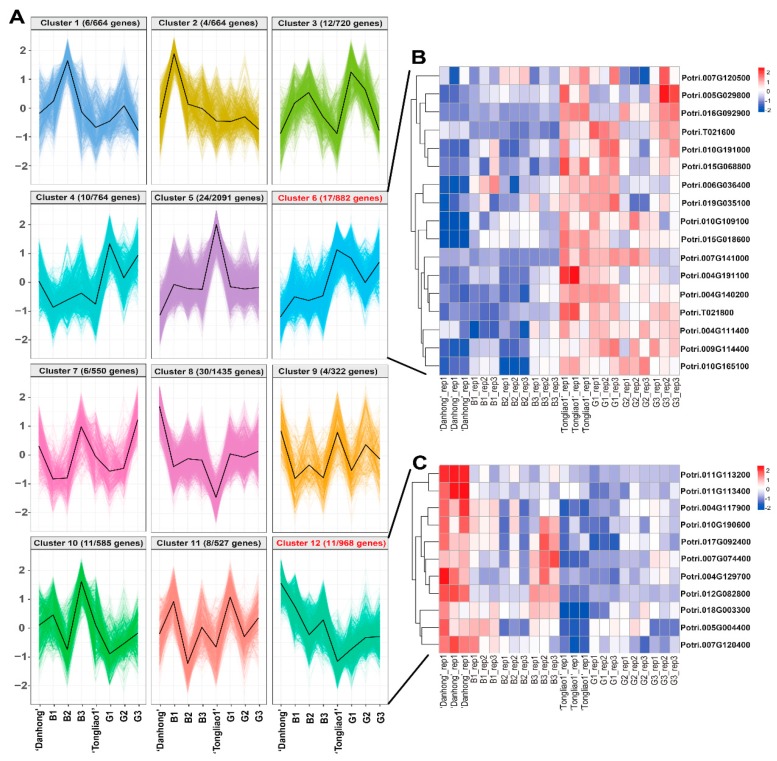
Cluster analysis of 10,172 DEGs based on the K-means method. (**A**) K-means clustering analysis identified 12 clusters of DEGs according to their expression patterns. The kilobase of transcript sequence per million (FPKM) of the DEGs in eight genotypes was transformed into Z-scores using an in-house Perl program. Mean expression values of each cluster were marked as black lines. (**B**) Heat map of 17 associated candidate genes in cluster 6. The Z-scores are color coded, as shown on the right color scale. (**C**) Heat map of 11 associated candidate genes in cluster 12. Z-scores are color coded, as shown on the right color scale.

K-means clustering was used to capture unique expression patterns of DEGs. The 10,172 DEGs were grouped into 12 clusters with different expression patterns (Figure 6A, Table S5). In cluster 6, the expression profiles of 882 DEGs were relatively lower in ‘Danhong’ and B group than ‘Tongliao1’ and G group. In contrast, the expression profiles of the cluster 12 (968 DEGs) were largely higher in ‘Danhong’ and B group than ‘Tongliao1’ and G group. Notably, 17 and 11 candidate genes in QTL intervals were identified in cluster 6 and cluster 12, respectively (Figure 6B,C). Among these overlapped genes, the homologous genes of *Potri.010G191000* and *Potri.004G111400* in *A. thaliana* were involved in root traits through transmembrane transporter activity [29]. Furthermore, the homologous gene of *Potri.010G190600* in *A. thaliana* encodes an l-tryptophan (Trp) biosynthesis enzyme, which regulates root development through mediating jasmonic acid-induced auxin accumulation and transport [30].

### 2.7. Identification of WGCNA Modules Associated with Phenotype Traits

**Figure 7:**
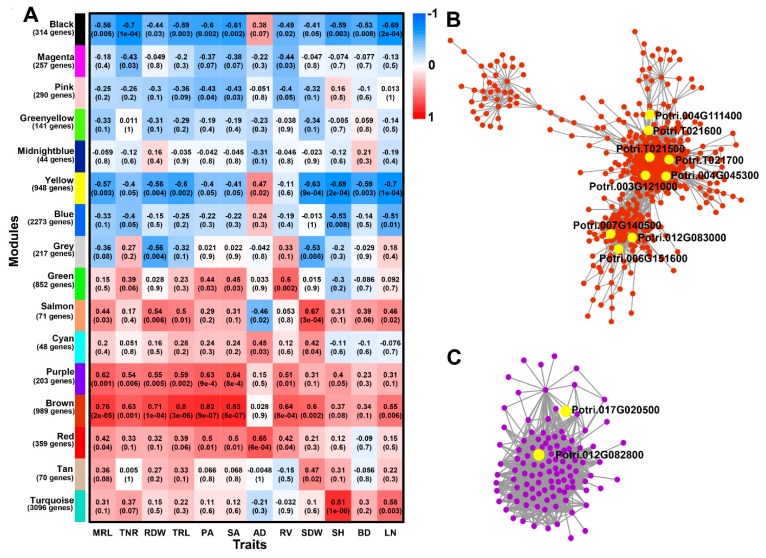
Weighted gene co-expression network analysis (WGCNA) of DEGs. (**A**) Module-trait relationships. Module-trait weight correlations and corresponding *p* values (in parenthesis). The left panel shows the 16 modules and the number of genes in each module. The color scale on the right shows module-trait correlation from −1 (blue) to 1 (red). (**B**) Cytoscape representation of co-expressed genes with edge weight ≥ 0.10 in “brown” module. (**C**) Cytoscape representation of co-expressed genes with edge weight ≥ 0.10 in “purple” module.

To reveal potential relationships between modules and traits, weighted gene co-expression network analysis (WGCNA) was performed with the 10,172 DEGs. Sixteen distinct modules were identified and labeled with different colors in a dendrogram (Figure S2). The sixteen modules exhibited different degrees of correlation with adventitious root and related shoot traits (Figure 7A). These results indicated that adventitious root and shoot development were under coordinated genetic basis control. The module brown of 989 DEGs and purple of 203 DEGs were all moderately and stably correlated with adventitious root traits including MRL, TNR, RDW, TRL, PA, SA, and RV (*r* > 0.50) (Table S8). Among them, nine and two candidate genes in QTL intervals were also identified in brown and pink modules, respectively. The correlation network of the brown and pink module genes with WGCNA edge weight > 0.10 were shown in Figure 7B,C. These overlapped genes also contained the potential root trait related gene *Potri.004G111400*. Besides, we also identified Potri.012G083000 in brown module. Its homologous gene in *Arabidopsis Crinkly* 4 (*ACR4*) has reported to form a ligand-receptor pair with *CLE40* to regulate *WOX5* expression for the balance between proliferation and differentiation in the shoot meristem [31].

### 2.8. Genes Involved Potentially in Regulating Adventitious Root and Related Shoot Traits

**Figure 8:**
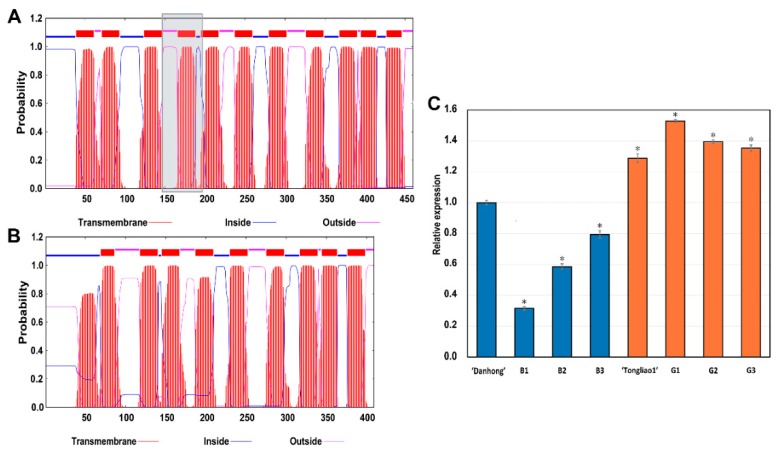
Protein functional domain and expression analysis of *PtAAAP19*. (**A**) Protein functional domain of *PtAAAP19* for ‘Danhong’. The grey box indicates the missing transmembrane domain in ‘Tongliao1’ from the 141st to 190th amino acid residue. (**B**) Protein functional domain of *PtAAAP19* for ‘Tongliao1’. (**C**) qRT-PCR analysis of *PtAAAP19* for eight genotypes in root tissue. Blue and orange color indicate poor-rooting genotypes and fine-rooting genotypes, respectively. Error bars represent the standard deviations, and * indicates significant differences compared with ‘Danhong’ at *p* < 0.05 level.

To identify potential function genes regulating adventitious root and related shoot traits, the genetic, genomic, and transcriptome analysis were integrated based on the results of QTL mapping, K-means clustering, and WGCNA. Three candidate genes *Potri.004G111400*, *Potri.T021600*, and *Potri.012G082800* were identified in all above analysis. The gene *PtAAAP19* (*Potri.004G111400*) encodes transmembrane amino acid transporter family protein [32], and its homologous gene in *A. thaliana* involves in adventitious root formation through the uptake of neutral and acidic amino acids in roots [33]. qRT-PCR analysis indicated that *PtAAAP19* exhibited higher expression levels in fine rooting genotypes (‘Tongliao1’, G1, G2, and G3) than poor rooting (‘Danhong’, B1, B2, and B3) (Figure 8C). This expression pattern confirmed the reliability of the RNA-Seq results and gene potential function. To reveal its genetic variations underlying differential expressions, we isolated the *PtAAAP19* gene from the two parents and found that a 153 bp deletion in its coding sequence (CDS) in ‘Tongliao1’ compared with ‘Danhong’. This CDS deletion resulted in the absence of a transmembrane domain in ‘Tongliao1’ from the 141st to 190th amino acid residue, which might affect the transmembrane amino acid transporter protein function (Figure 8A,B). These results might explain the different rooting ability between parents. However, further experiments need to in-depth investigate the detailed function of *PtAAAP19*.

## 3. Discussion

Adventitious root and related shoot traits are important quantitative traits for poplar growth and development. Their phenotypic variations are generally influenced by both genetic and environmental factors. Because the adventitious roots in soil culture were difficult to separate and measure, hydroponic culture was employed to rapidly collect clean and intact adventitious roots in our study. The F_1_ population exhibited extensive segregation variation for adventitious root and related shoot traits, indicating that the investigated traits were under quantitative inheritance regulation. These findings are in line with previous research [24]. The phenotypic traits were more tightly correlated within than between adventitious root and shoot traits. RV is the most reliable trait for distinguishing different genotypes because of its high variation and repeatability. The strong correlation was between PA and SA, confirming the logical relationship of adventitious root projected and surface area. Additionally, RV exhibited a highly significant correlation with PA and SA, indicating the utmost possible of common genetic components influencing these traits simultaneously.

A strong coupling growth between adventitious root and shoot has been reported in interspecific and intraspecific plant species [34]. The nature of coupling could be explained by the overall multi-genic and multi-locus [2]. Therefore, understanding the genetic basis of adventitious root and related shoot traits is crucial for breeding poplar genotypes with high biomass and reproduction capability. The high correlation among the investigated traits in this study indicated the existence of common QTLs controlling both adventitious root and related shoot traits. Indeed, we identified 10 QTL clusters associated with both adventitious root and shoot traits. Besides, the QTL loci within a QTL hotspot contribute cumulative phenotypic variation on a single trait. Forty QTL hotspots were detected on 14 LGs, which were likely to have large scale effects on a single trait. These results supported that adventitious root and shoot traits were under strongly coordinated genetic regulation. Similar studies have also been reported in *A. thaliana*, maize, and wheat [35,36,37]. The QTL cluster was more than the previous study in *P. trichocarpa × P. deltoides* [14]. The possible reason is that the hydroponic culture system had no mechanical impedance for adventitious root growth, which contributed to strong coordinate growth between the adventitious root and shoot [1]. Except for the common QTL clusters, unique QTL clusters were also detected to control either adventitious root traits or related shoot traits. This result suggested that variation in adventitious root traits did not totally rely on shoot traits, suggesting the existence of common and specific genetic regulatory mechanisms, being consistent with the previous hypothesis [19].

In our study, high repeatability was observed for adventitious root and related shoot traits, suggesting these traits were under strong genetic control. This finding was supported by a similar study of adventitious root and shoot regeneration ability traits in an F_2_ family of *P. trichocarpa* × *P. deltoides* [38]. A total of 233 QTLs controlling one or multiple traits were detected on 17 LGs, but these QTLs had no overlap with the previous investigations based on bi-parental mapping population [39,40]. The possible reasons might be the effect of different genetic background population, different markers used for genetic map construction, contrast map distance among different maps or contrast growing environments [41]. Therefore, it is critical and necessary to identify novel alleles for target biological traits with different genetic background materials and growth conditions.

To identify the potential genes responsible for the adventitious root and related shoot traits, DEGs from RNA-Seq data and candidate genes in QTL intervals were overlapped. A key gene *PtAAAP19* encoding amino acid transporters protein was found to have potential roles in root development because its homolog gene in *A. thaliana* transported amino acid across the cellular membrane in root phloem [42]. *P. trichocarpa* and *A. thaliana* diverged about 100–120 million years ago through ancient duplication events, which indicates that the orthologous pair genes originated from their common ancestor [43]. Thus, we speculated that *PtAAAP19* might play important roles in adventitious root formation and development. Previous research points out that a gene expression profile is a valuable clue for its functional study [44]. Furthermore, Street et al. [45] proposed that candidate genes can be selected by identifying genes with differential expression patterns between genotypes that possess extreme contrast target biological traits. The qRT-PCR results of *PtAAAP19* in root tissue of different genotypes with extreme contrast of rooting ability supported its potential role in regulating root traits. Although several evidences indicated the possible function of *PtAAAP19*, its specific functions and regulatory pathways need further verification in the future.

## 4. Materials and Methods

### 4.1. Plant Materials

A full-sib F_1_ population was originated from a cross between female *P. deltoides* ‘Danhong’ from Henan province and male *P. simonii* ‘Tongliao1’ from Inner Mongolia Autonomous Region. Fifteen-centimeter cuttings were produced from the two parents and 434 F_1_ genotypes, with six cuttings for each genotype. At least one robust bud was retained in each cutting. These cuttings were planted evenly in black plastic containers (48 × 38 × 12 cm), with 32 cuttings per container, and subsequently cultivated in hydroponic culture. The hydroponic water was changed every two days. The experiments were designed in a randomized block design with three blocks, with two cuttings of each genotype per block. The plants were incubated in the greenhouse of the Chinese Academy of Forestry under natural light conditions and temperature (20–25 °C).

### 4.2. Phenotypic Measurements

After cultivating the plants for two months, the phenotype traits of adventitious roots and shoots of all plants were measured. Root traits were scanned and analyzed using WinRHIZO Pro (Regent Instruments Inc, Quebec, QC, Canada), including total root length (TRL), projected area (PA), surface area (SA), average diameter (AD), and root volume (RV). Maximum root length (MRL) and shoot height (SH) were measured by a ruler, and basal diameter (BD) was measured by a vernier caliper. Total number of root (TNR) and leaf number of shoot (LN) were counted artificially. Following scanning and measuring, the root and shoot tissue were oven-dried at 80 °C for 48 h to measure root dry weight (RDW) and shoot dry weight (SDW).

### 4.3. Statistical Analysis

Analysis of block and clone variance was performed in SPSS21.0 software using the general linear model (IBM, Chicago, IL, USA). The statistical equation was Y_ijk_ =u + B_i_ + C_j_ + e_ijk_. Where Y_ijk_ was the phenotypic value for the jth clone in the ith block, u was the overall mean, B_i_ was the fixed effect of the ith block, C_j_ was the random effect of the jth clone, and e_ijk_ was the residual error. Repeatability was calculated using the variance parameter following the formula: R = ơ^2^_c_/(ơ^2^_c_ + ơ^2^_e_). Where ơ^2^_c_ and ơ^2^_e_ were the estimates of variances between-clone and within-clone, respectively [46].

The phenotypic data were analyzed using the SPSS v. 21.0 software to generate descriptive statistics, including the mean, minimum, maximum, standard deviation (SD), coefficient of variation (CV), skewness, and kurtosis. The skewness and kurtosis were used to estimate the frequency distribution normality [47]. The correlations among traits were analyzed using the Pearson correlation statistics coefficient. Phenotypic differences between two parents were checked using a paired *t*-test.

### 4.4. QTL mapping and Candidate Gene Analysis

A high-density genetic linkage map, including 5796 SNPs for 500 genotypes through whole-genome resequencing (being submitted in another paper), was used to detect QTL. QTL mapping for adventitious root and related shoot traits was performed using the multiple interval mapping (MIM) model with the MapQTL v. 6.0 software [48]. The logarithm of odds (LOD) threshold of 3.0 was chosen as evidence for the presence of QTLs. The QTL names started with an alphabet ‘q’, followed by an abbreviation of the trait and the linkage group (LG). The 20 kb upstream and downstream regions of the LOD peak position in the genome were regarded as target traits related genetic regulation locus, and the genes located within these genome regions were considered as the potential candidate genes. The function annotation of these candidate genes was searched in *P. trichocarpa* reference genome (https://phytozome.jgi.doe.gov/pz/portal.html).

### 4.5. RNA Isolation, Library Construction, and Illumina Sequencing

The two parents and six genotypes (G group including three genotypes G1-F405, G2-F66, and G3-F64 with fine-rooting ability; B group including three genotypes B1-F75, B2-F2, and B3-F196 with poor-rooting ability) were selected for RNA-Seq. Total RNA was isolated from the young adventitious root using a RNeasy Plant Mini Kit (Qiagen, Hilden, Germany), and DNase was treated with an RNase-Free DNase set (Qiagen, Hilden, Germany). The RNA quality and quantity were detected by 1% agarose gels, NanoPhotometer spectrophotometer, and Agilent 2100 bioanalyzer. Three biological replicates were performed for each genotype. A total of 24 RNA-Seq libraries were prepared as described previously [49] using NEBNext Ultra Directional RNA Library Prep Kit for Illumina (New England Biolabs, Miami, United States). The libraries were sequenced on an Illumina NovaSeq6000 platform and yielded 24 RNA-Seq datasets with a total of 123 million raw reads (Table S3). Quality control metrics were performed for raw reads using FastQC v. 0.11.7 software. The raw reads were filtered by removing reads containing adapter, poly-*N*, and low-quality reads using the NGS QC Toolkit [50]. The numbers of total reads and reads that were uniquely mapped to the genome were shown in Table S4.

### 4.6. Identification of Differentially Expressed Genes and K-Means Cluster Analysis

Transcript expression and differential expressed gene analysis of RNA-Seq data were carried out using HISAT v. 2.4 and DESeq2 v. 1.26.0. Differentially expressed genes (DEGs) were identified in each pairwise comparison set based on Baggerly’s test [51] with the expected number of fragments per kilobase of transcript sequence per million base pairs sequenced (FPKM). Genes with the |log2FoldChange| > 1.0 and false discovery rate (FDR) < 0.05 were considered differentially expressed. The FPKM values of DEGs were averaged across biological replicates and subsequently standardized to their Z-scores, which were performed in the K-means clustering algorithm. Heat map of candidate genes within the QTL region in cluster 6 and cluster 12 were plotted using heatmap packages in R software.

### 4.7. Weighted Gene Co-Expression Network Analysis

The co-expression network was analyzed using the weighted gene co-expression network analysis (WGCNA) in the R package. The network construction and module detection for DEGs were conducted using an unsigned type of topological overlap matrix (TOM) with a minimal module size of 30, and a merge cut height of 0.40 [52,53]. The different colors represented different modules. The relationship between modules and phenotypic data of adventitious root and related shoot traits was evaluated, and the co-expression networks of two significantly correlated modules (brown and purple) with WGCNA edge weight > 0.10 were represented using Cytoscape v. 3.1 [54].

### 4.8. Quantitative Real-Time PCR

Quantitative real-time PCR (qRT-PCR) analysis was adopted to measure the expression levels of the potentially associated genes in adventitious roots of parents and six genotypes that used for RNA-seq. First-strand cDNA synthesis was carried out with approximately 2 µg RNA using the SuperScript III reverse transcription kit (Life Technologies, USA) according to the manufacturer’s procedure. The primers were designed using Primer3 (http://frodo.wi.mit.edu/primer3/input.htm) with a melting temperature of 58–60 °C and production of 150–250 bp. The primers used in this study are listed in Table S9. qRT-PCR reaction system of 20 µL contained 2 µL of cDNA template, 10 µL of TB Green Premix Ex Taq (TaKaRa, Japan), 0.8 ul of forward primer, 0.8 µL of reverse primer, and 6.4 µL of ddH2O. qRT-PCR reaction was conducted on Roche LightCycler 480 Detection System with following protocol: pre-denature at 95 °C for 5 min; amplification of 45 cycles at 95 °C for 10 s, 60 °C for 10 s, and 72 °C for 10 s; melting curve analysis at 95 °C for 5 s and at 65 °C for 1 min; cooling at 40 °C for 30s. The *Ptactin* gene was used as a reference gene. All samples were performed in three biological replicates and four technique replicates.

### 4.9. Gene Cloning and Protein Structure Analysis

The genomic DNA of the two parents was extracted from fresh young leaves using the DNeasy plant Mini kit (Qiagen, German). Primer sequences for candidate gene coding sequences were designed in Primer 5.0 software (Table S10). PCR reactions were performed in a volume of 40 µL, containing 1 µL of genomic DNA, 20 µL of Primer Star mix (Takara, Japan), 17 µL of ddH_2_O, 1 µL of forward primer, and 1 µL of reverse primer. PCR amplification was performed using an Applied Biosystems instrument with the following protocol: 98 °C for 30 s, 38 cycles at 98 °C for 15 s, 56 °C for 15 s, and 68 °C for 90 s; and a final extension step at 68 °C for 6 min. DNAMAN was used to perform sequence alignment. The protein domains were analyzed using the TMHMM server 2.0 (http://www.cbs.dtu.dk/services/TMHMM).

## 5. Conclusions

In this study, we investigated the genetic architecture of adventitious root and related shoot traits of the F_1_ population originating from a cross between *P. deltoides* ‘Danhong’ × *P. simonii* ‘Tongliao1’. All investigated traits showed extensive segregation, high repeatability, and significant correlation, indicating these traits were under quantitative genetic regulation. QTL mapping identified 233 QTLs associated with the adventitious root and related shoot traits, explaining 3.1%–19.8% of phenotypic variation, demonstrating that the coupling growth between adventitious root and shoot was under multi genetic control. Ten QTL clusters responsible for both adventitious root and related shoot traits and 15 QTL clusters regulating the adventitious root individually or shoot traits manifested the existence of common and specific genetic regulatory mechanisms. Combining the QTL and transcriptome analysis, three associated genes (*Potri.004G111400*, *Potri.T021600*, and *Potri.012G082800*) within the QTL intervals were differentially expressed between fine-rooted and poor-rooted genotypes. Especially, *Potri.004G111400* (*PtAAAP19*) was regarded as a potential function gene for adventitious root traits through its differential expression profiles and protein domain variation. Our results dissected the genetic basis of investigated traits and provided potential genetic resources for biotechnological breeding of elite varieties in forest trees.

## Figures and Tables

**Table 1:** Phenotype analysis of parents and F_1_ population.

Trait	Parents		F_1_ population						
	*P. deltoides* ‘Danhong’	*P. simonii* ‘Tongliao1’	Min	Max	Mean ± SD	CV	Kurtosis	Skewness	Repeatability
Maximum root length (MRL)/cm	4.10	11.93 **	2.910	22.200	12.74 ± 3.12	0.245	0.361	−0.231	0.84
Total root length (TNR)/n	2.00	11.00 **	1.000	21.000	8 ± 3.74	0.442	0.146	0.557	0.89
Root dry weight (RDW)/g	0.02	0.05 **	0.005	0.170	0.06 ± 0.03	0.563	−0.017	0.724	0.81
Total number of root (TRL)/cm	22.30	88.31 **	3.111	347.042	135.94 ± 65.96	0.485	−0.145	0.325	0.84
Projected area (PA)/cm^2^	2.46	6.30 **	0.061	37.999	12.32 ± 6.20	0.503	0.533	0.700	0.89
Surface area (SA)/cm^2^	7.74	19.81 **	0.193	102.960	38.50 ± 19.15	0.497	0.094	0.595	0.89
Average diameter (AD)/mm	0.53	0.34	0.089	1.025	0.47 ± 0.18	0.388	0.255	0.921	0.76
Root volume (RV)/cm^3^	0.04	0.07 **	0.010	0.496	0.19 ± 0.13	0.697	−0.325	0.962	0.88
Shoot dry weight (SDW)/g	0.23	0.26	0.010	0.753	0.25 ± 0.14	0.559	0.387	0.667	0.86
Shoot height (SH)/cm	4.77	20.33 **	0.65	16.17	7.13 ± 3.35	0.470	−0.634	0.191	0.84
Basal diameter (BD)/mm	2.20	2.45	1.05	2.84	1.94 ± 0.30	0.153	0.011	−0.068	0.72
Leaf number of shoot (LN)/n	4.00	9.00 **	2.00	13.00	7 ± 2.21	0.32	−0.562	−0.015	0.87

**—the difference between *P. deltoides* ‘Danhong’ and *P. simonii* ‘Tongliao1’ at *P* < 0.01; CV—coefficient variation.

**Table 2:** Quantitative trait locus (QTL) clusters for the investigated traits in the F_1_ population.

QTL Clusters	Location/cM	Marker	QTLs
LG1	61.517	np1680	qMRL-LG1-2; qRDW-LG1-4
LG3	94.464	lm5516	qAD-LG3-1; qRV-LG3-1
LG5	108.269	np5635/np3887	qRV-LG5-1; qTNR-LG5-5 to 6; qPA-LG5-1
LG5	109.465	lm4991	qTNR-LG5-7; qPA-LG5-2
LG5	144.026	lm1039	qRV-LG5-2; qPA-LG5-3
LG5	145.220	np3795	qRV-LG5-3; qPA-LG5-4
LG5	145.598	lm4903	qRV-LG5-4; qPA-LG5-5
LG5	146.390	lm1038	qRV-LG5-5; qPA-LG5-6; qSA-LG5-1
LG5	146.547	lm4886/lm4889/lm4897/lm4895	qRV-LG5-7 to 10; qPA-LG5-7 to 10; qTNR-LG5-13 to 14; qSA-LG5-2
LG5	150.040	hk421/np2628/np3744/np5403	qRV-LG5-9 to 12; qPA-LG5-11
LG8	113.645	np5178	qTNR-LG8-1; qRDW-LG8-1
LG8	114.080	lm4584/lm6091	qTNR-LG8-2; qRDW-LG8-2 to 3
LG9	181.569	lm6429/hk339	qTNR-LG9-3; qLN-LG9-32
LG9	16.719	hk321	qSH-LG9-1; qBD-LG9-1
LG11	1.838	lm1575	qAD-LG11-1; qRV-LG11-1
LG14	93.110	lm2588	qMRL-LG14-7; qTNR-LG14-1 to 2; qRDW-LG14-1
LG18	61.454	hk178	qRDW-LG18-2; qLN-LG18-1
LG18	63.535	lm1912	qRDW-LG18-3; qLN-LG18-2
LG18	63.546	hk181	qRDW-LG18-4; qLN-LG18-3
LG18	63.554	lm1961	qRDW-LG18-5; qLN-LG18-4
LG18	103.390	np1865	qRDW-LG18-11; qLN-LG18-5
LG18	103.474	np2428	qRDW-LG18-12; qLN-LG18-6
LG18	104.882	np2706	qRDW-LG18-17; qLN-LG18-7
LG18	108.381	hk190	qRDW-LG18-22; qLN-LG18-8
LG18	120.773	np1530	qRDW-LG18-27; qLN-LG18-13

**Table 3:** QTL hotspots for the investigated traits in the F_1_ population.

QTL Hotspots	Location/cM	QTLs
LG1-hotspot	60.187–61.536	qMRL-LG1-1 to qMRL-LG1-3
LG1-hotspot	111.46–111.724	qTNR-LG1-1 to qTNR-LG1-3
LG1-hotspot	60.826–78.527	qRDW-LG1-1 to qRDW-LG1-6
LG1-hotspot	110.921	qAD-LG1-1; qAD-LG1-2
LG3-hotspot	94.464–97.935	qRV-LG3-1 to qRV-LG3-4
LG5-hotspot	83.835–94.992	qTNR-LG5-1 to qTNR-LG5-4
LG5-hotspot	108.269–120.75	qTNR-LG5-5 to qTNR-LG5-12
LG5-hotspot	146.547–151.667	qTNR-LG5-13 to qTNR-LG5-16
LG5-hotspot	108.269–109.465	qPA-LG5-1; qPA-LG5-2
LG5-hotspot	144.026–146.547	qPA-LG5-3 to qPA-LG5-11
LG5-hotspot	146.39–146.547	qSA-LG5-1; qSA-LG5-2
LG5-hotspot	108.269–116.987	qRV-LG5-1; qRV-LG5-2
LG5-hotspot	144.026–150.04	qRV-LG5-3 to qRV-LG5-14
LG6-hotspot	48.932–48.967	qTNR-LG6-1; qTNR-LG6-2
LG8-hotspot	136.892	qMRL-LG8-1; qMRL-LG8-2
LG8-hotspot	113.645–114.08	qTNR-LG8-1; qTNR-LG8-2
LG8-hotspot	113.645–115.205	qRDW-LG8-1 to qRDW-LG8-6
LG8-hotspot	96.906–99.318	qAD-LG8-1 to qAD-LG8-5
LG9-hotspot	16.194–161.708	qTNR-LG9-1; qTNR-LG9-2
LG9-hotspot	181.569–181.707	qTNR-LG9-3; qTNR-LG9-4
LG9-hotspot	1.005–5.783	qLN-LG9-1 to qLN-LG9-6
LG9-hotspot	39.995–59.604	qLN-LG9-7 to qLN-LG9-19
LG9-hotspot	60.121–64.262	qLN-LG9-20 to qLN-LG9-26
LG9-hotspot	128.667–137.152	qLN-LG9-27 to qLN-LG9-29
LG9-hotspot	162.57–181.569	qLN-LG9-30 to qLN-LG9-32
LG10-hotspot	48.46–52.626	qLN-LG10-1 to qLN-LG10-5
LG11-hotspot	88.512	qSH-LG11-1; qSH-LG11-2
LG14-hotspot	82.879–93.11	qMRL-LG14-1 to qMRL-LG14-7
LG14-hotspot	93.11–111.965	qRDW-LG14-1 to qRDW-LG14-7
LG14-hotspot	96.276–98.586	qBD-LG14-1 to qBD-LG14-6
LG15-hotspot	66.466	qSDW-LG15-1; qSDW-LG15-2
LG16-hotspot	70.358–80.56	qTNR-LG16-1 to qTNR-LG16-6
LG17-hotspot	75.914–89.987	qTNR-LG17-1 to qTNR-LG17-7
LG18-hotspot	58.039–65.532	qRDW-LG18-1 to qRDW-LG18-6
LG18-hotspot	79.845–97.932	qRDW-LG18-7 to qRDW-LG18-10
LG18-hotspot	103.39–121.726	qRDW-LG18-11 to qRDW-LG18-29
LG18-hotspot	61.454–63.554	qLN-LG18-1 to qLN-LG18-4
LG18-hotspot	103.39–120.773	qLN-LG18-5 to qLN-LG18-13
LG19-hotspot	107.005–110.402	qSDW-LG19-1; qSDW-LG19-2
LG19-hotspot	103.889–107.006	qBD-LG19-1 to qBD-LG19-3

## Supplementary Materials

Supplementary materials can be found at https://www.mdpi.com/1422-0067/20/24/6114/s1. Figure S1. (A) Heat map showing the Pearson’s correlation coefficient of pairwise comparison between samples. (B) Principal component analysis (PCA) showing the uniformity among three replicates and the relationships of eight genotypes. Figure S2. Hierarchical cluster tree showing 16 modules of co-expressed genes. Each of the 10,172 DEGs was represented by a leaf in the tree, and each of the 16 modules by a major tree branch. The lower panel shows modules in designated colors. Table S1. ANOVA analysis of adventitious root and related shoot traits in the F_1_ population. Table S2. Annotation of candidate genes associated with the adventitious root and related shoot traits. Table S3. Overview of RNA-Seq data. Table S4. Mapping characterization of RNA-Seq data for each genotype. Table S5. DEGs in 21 pairwise comparison sets. Table S6. Information on differentially expressed DEGs and TFs between 21 pairwise comparison sets. Table S7. Summary of overlapped genes between QTL and RNA-Seq analysis. Table S8. Module genes of WGCNA analysis. Table S9. Primers for qRT-PCR. Table S10. Primers for *PtAAAP19* coding sequence in ‘Danhong’ and ‘Tongliao1’.
