# Supplementary figures and images for: Deciphering Genetic Architecture of Adventitious Root and Related Shoot Traits in Populus Using QTL Mapping and RNA-Seq Data
Source: Int J Mol Sci. 2019 Dec 4;20(24):6114. doi: 10.3390/ijms20246114 (PMC6941115; doi:10.3390/ijms20246114)

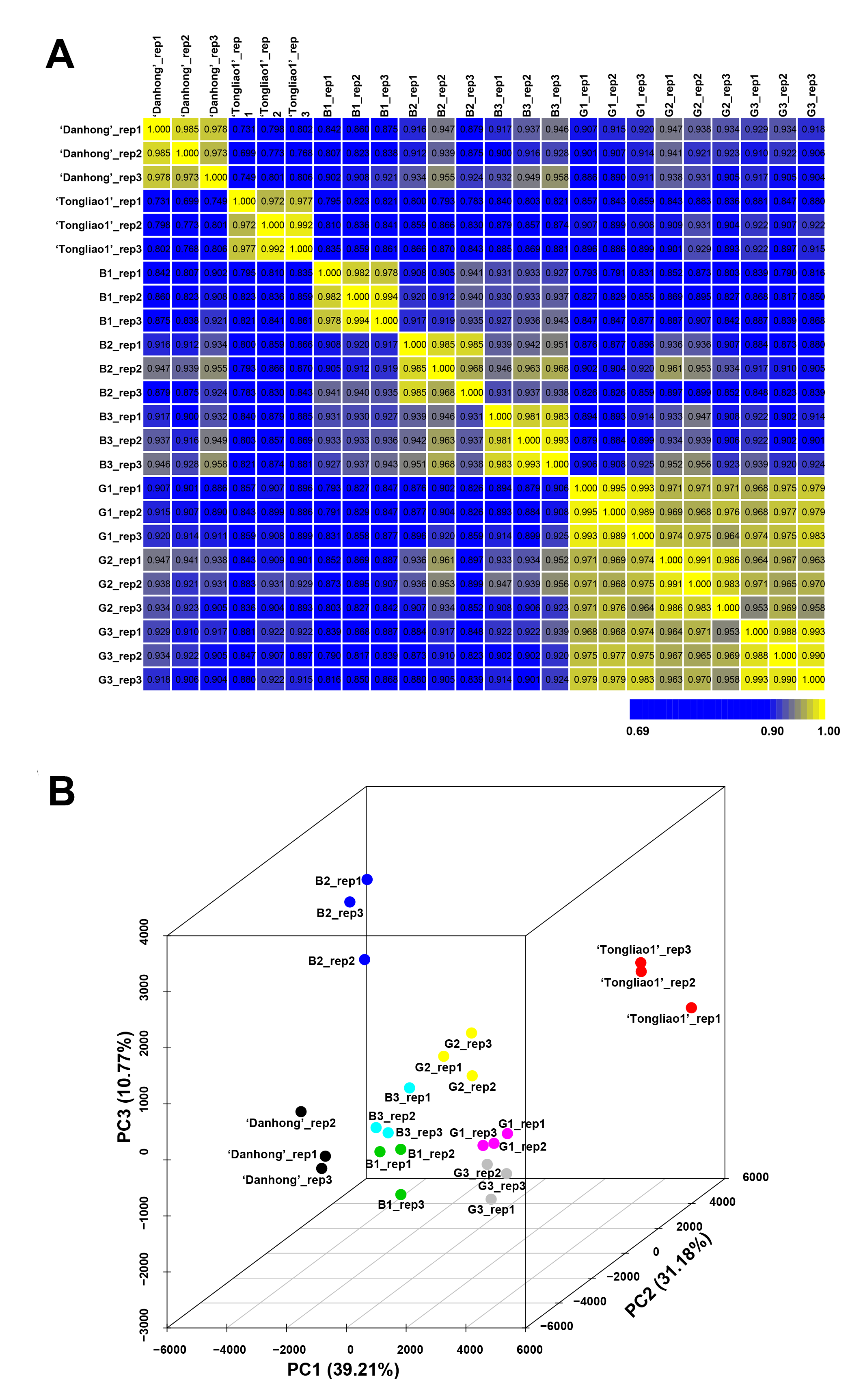

Supplement: Supplementary file 1 [file ijms-20-06114-s001.zip › Figure S1.tiff]

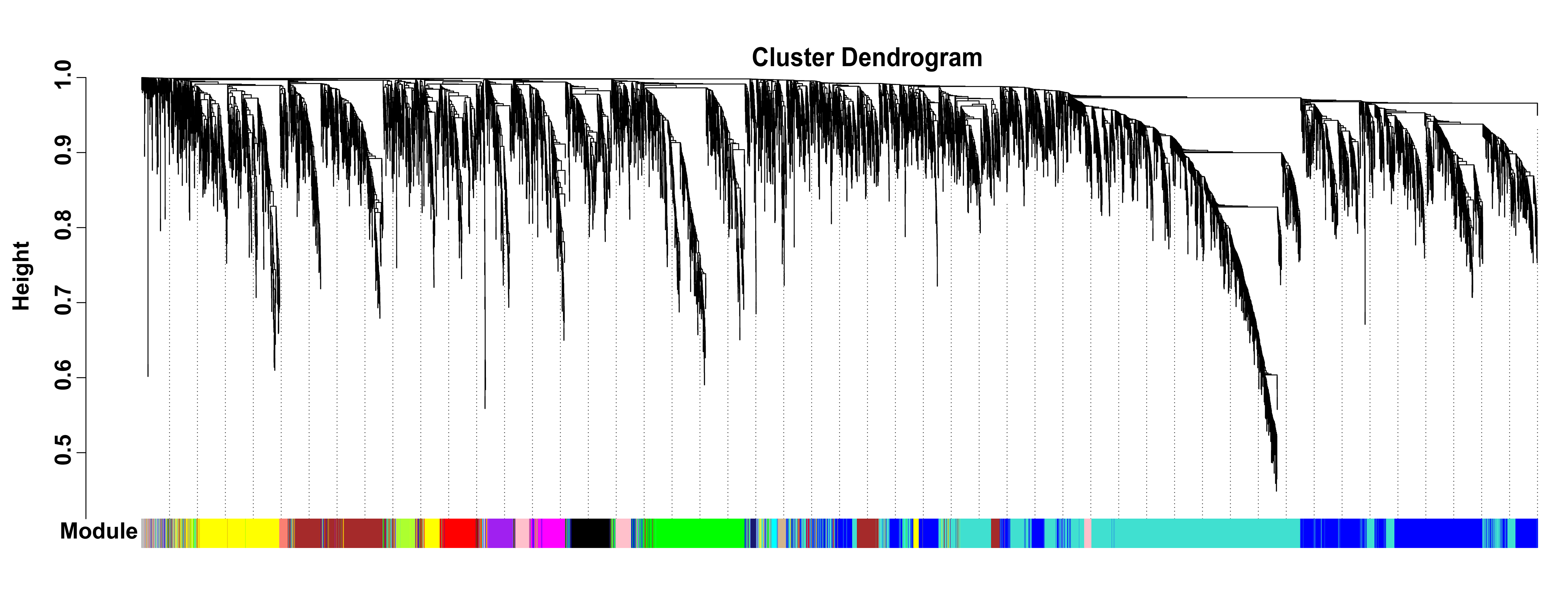

Supplement: Supplementary file 1 [file ijms-20-06114-s001.zip › Figure S2.tiff]
